# TopoQual polishes circular consensus sequencing data and accurately predicts quality scores

**DOI:** 10.1186/s12859-024-06020-0

**Published:** 2025-01-16

**Authors:** Minindu Weerakoon, Sangjin Lee, Emily Mitchell, Haynes Heaton

**Affiliations:** 1 Auburn University, Auburn, AL 36849 USA; 2 Wellcome Sanger Institute, Wellcome Genome Campus, Hinxton, CB10 1SA UK; 3 Wellcome-MRC Cambridge Stem Cell Institute, Cambridge Biomedical Campus, Cambridge, UK; 4 Department of Haematology, University of Cambridge, Cambridge, UK

**Keywords:** Topoqual, Deep consensus, Pacbio, Circular consensus sequencing, High fidelity, Somatic mutations, Quality scores, Error correcting

## Abstract

**Background:**

Pacific Biosciences (PacBio) circular consensus sequencing (CCS), also known as high fidelity (HiFi) technology, has revolutionized modern genomics by producing long (10 + kb) and highly accurate reads. This is achieved by sequencing circularized DNA molecules multiple times and combining them into a consensus sequence. Currently, the accuracy and quality value estimation provided by HiFi technology are more than sufficient for applications such as genome assembly and germline variant calling. However, there are limitations in the accuracy of the estimated quality scores when it comes to somatic variant calling on single reads.

**Results:**

To address the challenge of inaccurate quality scores for somatic variant calling, we introduce TopoQual, a novel tool designed to enhance the accuracy of base quality predictions. TopoQual leverages techniques including partial order alignments (POA), topologically parallel bases, and deep learning algorithms to polish consensus sequences. Our results demonstrate that TopoQual corrects approximately 31.9% of errors in PacBio consensus sequences. Additionally, it validates base qualities up to q59, which corresponds to one error in 0.9 million bases. These improvements will significantly enhance the reliability of somatic variant calling using HiFi data.

**Conclusion:**

TopoQual represents a significant advancement in genomics by improving the accuracy of base quality predictions for PacBio HiFi sequencing data. By correcting a substantial proportion of errors and achieving high base quality validation, TopoQual enables confident and accurate somatic variant calling. This tool not only addresses a critical limitation of current HiFi technology but also opens new possibilities for precise genomic analysis in various research and clinical applications.

**Supplementary Information:**

The online version contains supplementary material available at 10.1186/s12859-024-06020-0.

## Background

Somatic variants, unlike germline variants, occur in a subset of cells. The fraction of cells in which a given somatic variant occurs affects our ability to sample it. And in order to be confident it is a true positive rather than an erroneous base, we often must sample it multiple times [1–3]. In order to confidently call a somatic variant from a single DNA read, the error rate of that base must be significantly lower than the rate of somatic variants expected in that genome [4, 5].

In order to have high base accuracy, we must create a high signal to noise ratio (SNR) system. The size of a single nucleotide of DNA is smaller than the wavelength of light. This makes measuring the sequence of nucleotides of a single DNA molecule optically near the theoretical diffraction limit of detection [6, 7]. To overcome this, historically each molecule was amplified by cloning, polymerase chain reaction (PCR) [8], or bridge amplification [9]. This increases the SNR by measuring thousands or millions of nucleotides instead of a single one. However, amplification methods are not error-free and if an error occurs early on in these systems, the vast majority of molecules will have the erroneous nucleotide and we will confidently sequence this error [10, 11] putting a cap on the theoretical base accuracy.

In addition to this limitation, ever since Solexa introduced the Genome Analyzer in the early 2000s, the progression of DNA sequencing technologies has focused on data throughput over data quality. Data quality is, of course, multifactorial. For DNA sequencing it is a combination of the base level accuracy as well as the length of the read. In fact, the length of the sequence increases the theoretical information content exponentially while the base accuracy of each base does so only linearly. There are many repeats in genomes caused by a multitude of phenomena including but not limited to transposable elements, a variety of duplication events [12], and viral inserts [13]. To resolve a sequence, we must have reads greater than the length of these repeats in order to anchor on unique sequences [14]. In 2010, PacBio introduced their continuous long read (CLR) technology using sophisticated zero mode waveguides (ZMW) to limit the number of nucleotides in the detection space thus increasing the resolution very near the diffraction limit of light [6]. Over the next decade, they improved the processivity of their DNA polymerase enzyme as well as detector and software to create reads that were tens of kilobases long. This is in contrast to Illumina reads which were ~ 100–150 bases. The downside to this technology was that the bases had a high error rate (85–90% accurate) [15]. While the theoretical information content of these reads were very high due to their length, they were very challenging to work with computationally as they relied on extensive all-vs-all alignment and multiple alignment rather than fast exact-match seeding [14, 16].

More recently, Pacbio released their CCS/HiFi technology. In this technology, they attach a hairpin adapter to each end of the molecule creating a circular construct. Using strand-displacing DNA polymerase, they are able to sequence the full circular construct multiple times [7]. They then separate each subread of the forward and reverse strand and create a consensus sequence from them. This, along with improvements in DNA polymerase processivity allow for many passes on the same circular molecule. Because the errors on each sequencing pass on the molecule are largely independent of each other, the accuracy of the consensus sequence is largely only theoretically limited by the number of passes and our ability to create accurate multiple alignments of these subreads. This creates long (15 kb +) and highly accurate (99.9 + %) reads with some bases reaching a theoretical accuracy on the order of 1–10^−9^ or higher assuming no upstream error sources, optimal multiple alignment, and truly independent measurements. This technology has revolutionized genome assembly [17–19], structural variant analysis [20], and other aspects of genomics. However, when Pacbio estimates the quality of each base, it gives many bases a Phred scaled quality of 93 (corresponding to an accuracy of 1–10^−9.5^) and when compared to a sample with nearly perfect ground truth knowledge, these bases only validate at q45. Therefore, we cannot trust these quality score estimates. It is our goal here, to create a system which not only estimates these quality scores accurately, but also can correct some of the errors in the consensus base calling algorithms currently used. While there are many other HIFI base correcting algorithms such as Hifiasm [17], HiCanu [21], Verkko [22], mdbg [23], and LJA [24], these are based on correcting reads based on other reads in the sample. This will “correct” aka remove true somatic variants that are an important part of understanding the biology of these samples. Therefore, we only compare our base correction to Deepconsensus [25], the primary other tool used for base correction without removal of somatic variants. This will pave the way to allow for accurate somatic mutation calling with CCS data even when the somatic variant is only sampled by a single read in the sample.

Current long read somatic mutation detection algorithms, such as ClairS [26] and DeepSomatic [27], rely on deep learning models to improve the accuracy of identifying mutations in genomic data. These models are designed to learn complex patterns from sequencing data, allowing for enhanced detection of somatic mutations, including those in noisy or low-coverage regions. These algorithms do not require the exact base quality scores, which represent the confidence in the accuracy of each base call for their processing; instead, they only need relative quality scores, as the models are designed to interpret and adjust these values automatically to enhance somatic variant detection. To develop a deterministic algorithm for long-read sequencing, such as those used in short-read tools like Mutect2 [5] or Strelka2 [28], precise base quality scores are crucial. Even in short-read sequencing, base quality scores must be recalibrated for somatic variant detection, as the raw scores often do not accurately reflect the true quality of the data, potentially leading to errors if not adjusted properly [29, 30].

### Evidence for overestimation of base quality scores

Current HIFI data gives most bases a quality value of 93 corresponding to an error rate of 5e-10 or 1 error in 2 billion bases. In order to validate these quality scores, we compared quality scores on bases that were different from the reference but also not germline variant locations. These remaining mismatches should either be somatic variants or errors. We tested this vs data generated from umbilical cord blood which should have an exceedingly low number of true somatic variants due to its relatively young age with an expected number of somatic mutations of 236 (see supplement for details). In practice, we identified approximately 33,436,615,032 bases with an original quality score of 93, among which there were 934,358 mismatches. This results in an effective base quality score of only 45, indicating that the true confidence in the accuracy of these bases is much lower than the reported quality score. This discrepancy underscores the notion that the initial quality scores assigned to PacBio HiFi reads do not accurately reflect the actual error rates, thus supporting our claim that these sequences overestimate their base qualities (for example somatic variant detection calculations see supplement 3.4).

### Implementation

See Fig. 1.

**Fig. 1:**
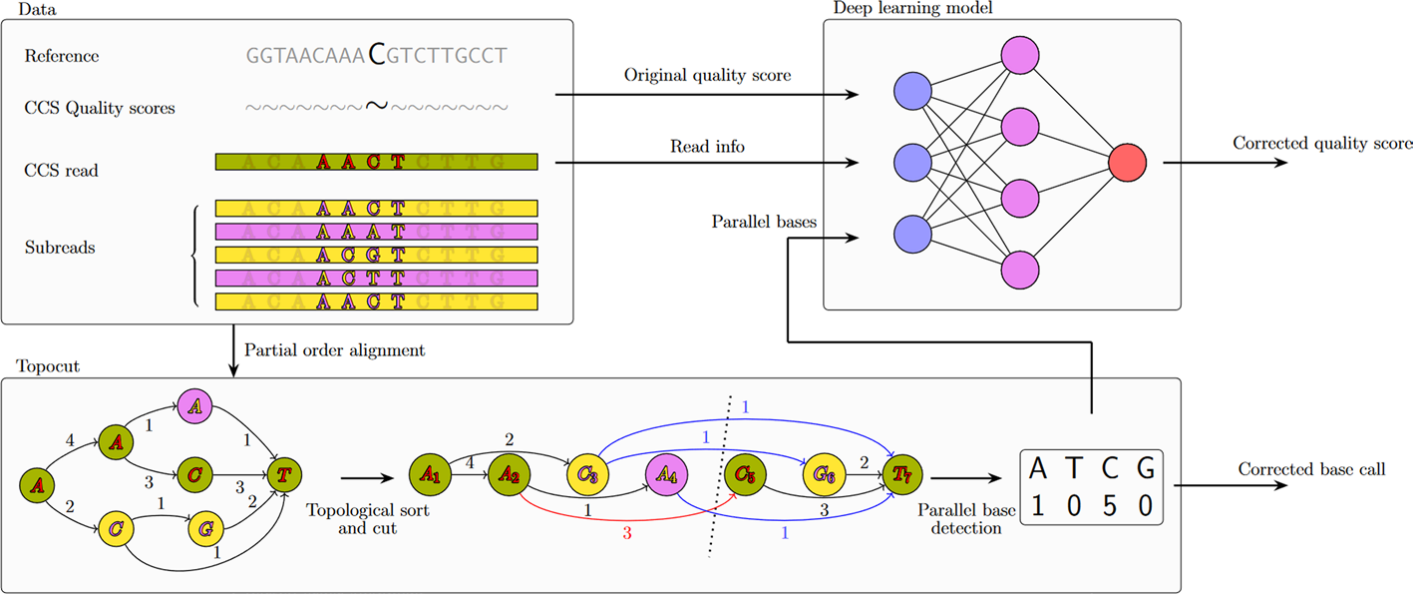
The quality score estimation strategy of topoqual. Subreads are aligned together with the current consensus sequence. Then potential alternative bases in the multiple alignment are detected via finding parallel bases in the POA graph. These along with multiple other signals are sent to a deep learning system to learn a quality score estimator

### CCS library preparation and sequencing

Umbilical blood from a newborn female was collected in 40–60 mL lithium-heparin tubes, and processed for blood granulocyte isolation using Lymphoprep. High molecular weight (HMW) DNA was extracted from the granulocytes using the Qiagen MagAttract HMW DNA extraction kit (67,563) and sheared into 16–20 kb DNA fragments using the Megaruptor 3 system (B06010003) with a speed setting of 30. CCS sequencing libraries were then prepared following the standard CCS library preparation protocol 1.0 (100–222-300), and sequenced on Sequel IIe instruments at the Wellcome Sanger Institute.

### Overview

We present TopoQual, a tool for polishing the sequences and providing precise base quality scores through the utilization of parallel (potential alternative) bases. The workflow of TopoQual is illustrated in (Fig. 1). To begin, we perform POA multiple alignment of subreads with the current consensus. We then use our algorithm (topocut) to find the parallel bases of the calling base in the POA graph. These parallel bases from topocut are used to correct the original base call if an alternate base has a higher count than the original base. Additionally, the parallel bases, in conjunction with the trinucleotide sequence of the read, and the target base’s quality score are input to the deep learning model which outputs a corrected quality score. During training, the deep learning model treats mismatch bases that are not a germline mutation as errors because the number of somatic mutations in our umbilical cord blood data is expected to be much smaller than the number of errors observed. While the reference genome is necessary for the training of this model, it is not required for new datasets which can be corrected and base quality recalibrated with just the subreads.

### Topocut

The partial order alignment data structure [31], which is a graph containing rich details about the aligned sequence structure, allows us to analyze the alternate pathways from the target base’s path; we define these alternate pathways as parallel bases. We use partial order alignment as it guarantees the optimal alignment of a new sequence versus the sequences already aligned. How Partial order alignment works is by extending standard dynamic programming sequence alignment [32, 33] to work with partial order graphs adding a sequence to the graph in each step.

TopoCut is the algorithm we used to procure parallel bases from the partial order alignment graph in our tool TopoQual. To accurately find the parallel bases, TopoCut first does partial order multiple sequence alignment with the CCS read and then the subreads. This outputs a partially ordered graph in which sequence letters are represented by nodes, and number of agreeing sequences are represented by edge weights. Then we sort nodes in a topological fashion and rank them according to the order. In this sorted graph, TopoCut makes a cut in front of the calling base and identifies the edges that intersect this cut, these edges are what we considered the parallel bases.

In our example (Fig. 1), we aim to find the parallel bases of calling base C, which has a topological ranking of 5. First, the parent edge weight of the calling base C is added to parallel base count. Then, parent–child rank pairs which sandwich the calling base C are discovered (3–6, 3–7, 4–7), and corresponding edge weights are added to the parallel base count to get the final parallel base count [A = 1, C = 5, G = 0, T = 0]. Total parallel base count is 6 which agrees with the total number of sequences, therefore further action is needed.

TOPOCUT_IDENTIFY_PARALLEL_BASES() (Algorithm 1) accomplishes the above by, adding the calling base’s weight in graph to the parallel bases array (line 2) and adding the corresponding parent edge weights if there are any parent–child rank pairs that sandwich the calling base’s rank (line 3–7). If the count of parallel bases does not sum up to the total number of sequences Num (line 8 −9), the process is done in reverse (line 9–20).

**Figure:**
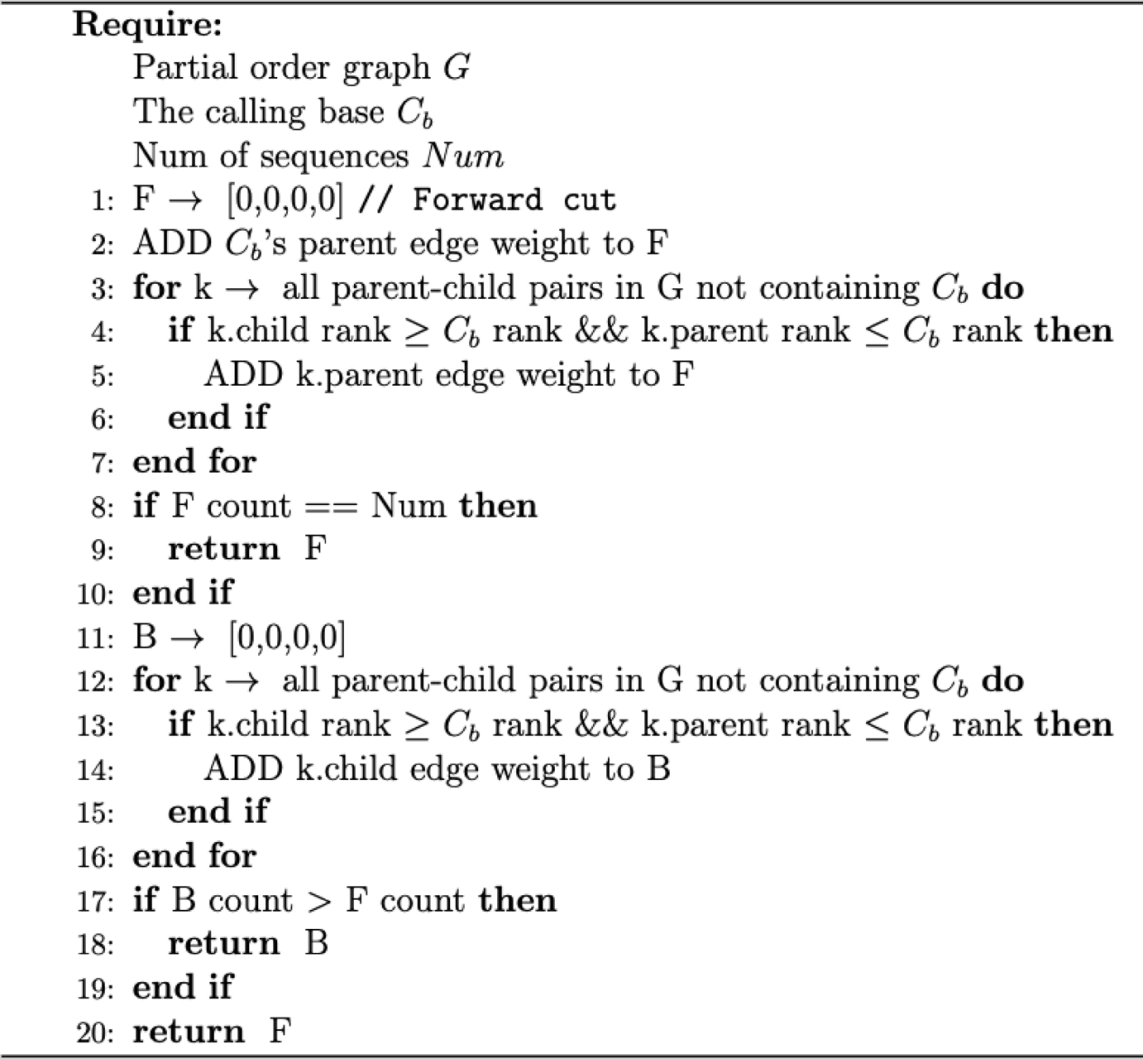
Algorithm 1. Identify parallel bases

### Deep learning model

The deep learning model is at the core of topoqual which takes in various information about the read and outputs the predicted quality score. Inputs to the model encompass the trinucleotide sequence of the read, Pacbio CCS quality score, parallel bases by topocut, average inter pulse duration, average pulse width, and the signal to noise ratio of the bases [34]. During the training phase, a dataset with labels of 0 for a reference mismatched base and 1 otherwise is utilized. Further details in the supplementary.

## Results

### Validation data

To validate our methodology, we sequenced a cord blood sample with few somatic mutations (40–50 somatic substitutions per cell [35]) from a 9-month-old female donor giving an expected number of somatic mutations in our 30 × data of 675. Given the low mutation burden of this sample, most of the mismatches between the sample and the reference genome (524,575 observed) is a result of either library or sequencing errors, and not somatic mutations, indicating that the majority of these occurrences are likely attributed to erroneous base calls.

### Sequence polishing

**Fig. 2:**
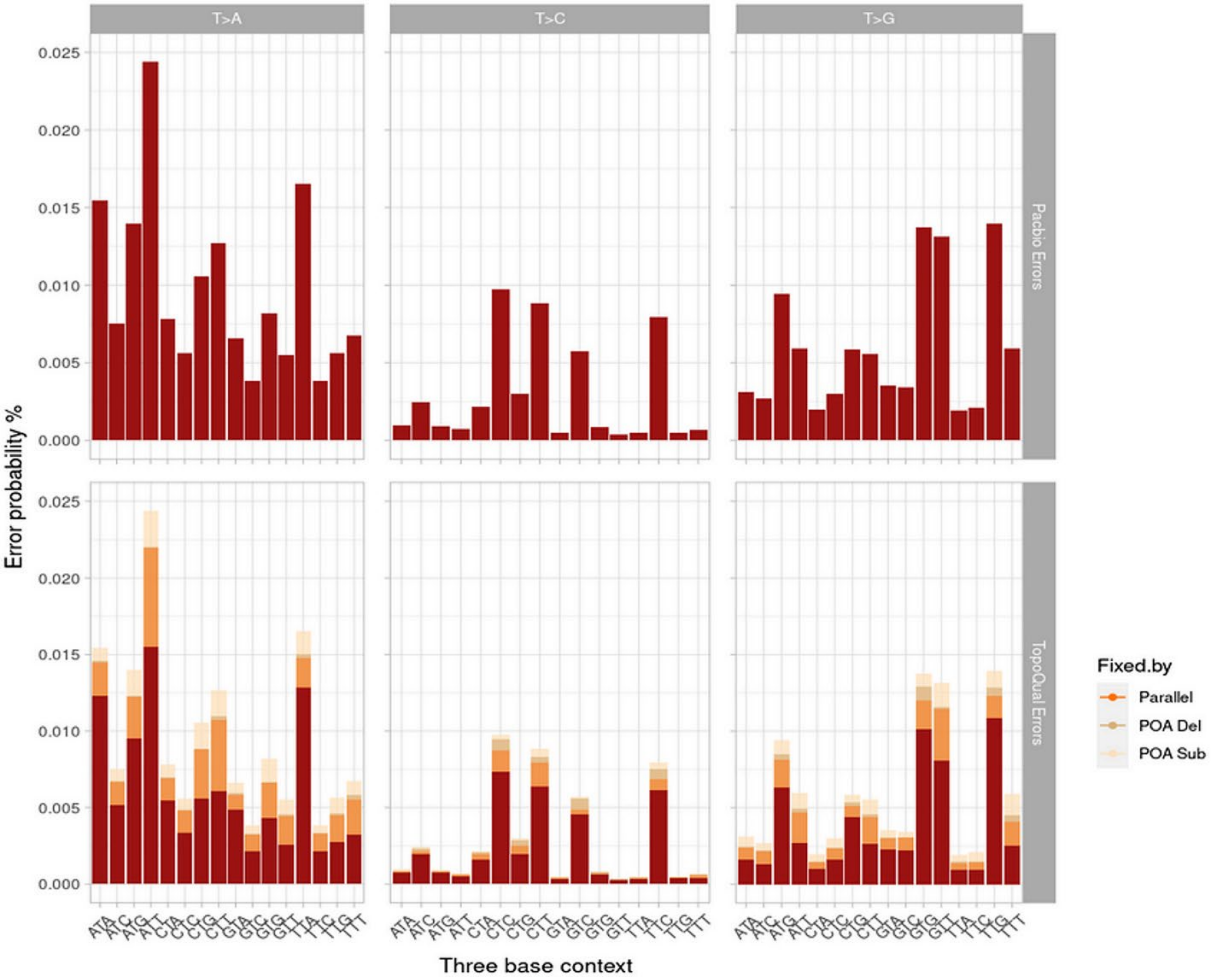
Errors present before (top) and after polishing (bottom) by topoqual in the validation dataset T > X for our 3 types of corrections (parallel bases prefer a different base, POA consensus deletes a base, and POA consensus substitutes a base), chr2

Topoqual conducts partial order alignment using PacBio CCS reads and their subreads to obtain parallel bases. Within this process, various mismatches (errors) with the reference are corrected using different techniques (parallel bases prefer a different base, POA consensus deletes a base, and POA consensus substitutes a base). (Fig. 2) illustrates the polishing of T > X mutations with respect to the three-base context. The sensitivity and specificity of sequence polishing are 31.9% and 99.6% respectively. The percentages of errors corrected in different steps are as follows (Table 1):

**Table 1:** Percentage of errors corrected by topoqual using different techniques

Polished by method	Number of bases polished (% of errors)
POA SUB	166 Kb (9%)
POA DEL	66 Kb (3.5%)
PARALLEL	361 Kb (19.5%)
TOPOQUAL	594 Kb (31.9%)

Total number of bases analyzed 11 Gb, errors present 1.8 Mb

The (Table 2) compares the error rates and maximum quality scores from validation dataset (Max Q) of sequencing data for PacBio, Deep Consensus, and TopoQual across several chromosomes (Chr2, Chr3, Chr4, Chr18, Chr19, Chr20, Chr21). For each chromosome, TopoQual consistently exhibits lower error rates and higher Max Q scores than the other methods. Specifically, TopoQual achieves higher max Q for all chromosomes except Chr4 and archives ~ 0.06% lower error rates for all chromosomes. These improvements demonstrate TopoQual's superior performance in reducing errors and enhancing base quality scores compared to PacBio and Deep Consensus.

**Table 2:** Results of different methods on the validation dataset

Chromosome	Pacbio			Deep consensus			Topoqual		
	Error/Total base pairs	Error rate	Max Q	Error/Total base pairs	Error rate	Max Q	Error/Total base pairs	Error rate	Max Q
Chr2	562 Kb/3.3 Gb	0.017%	46	585 Kb/3.8 Gb	0.015%	49	263 Kb/2.7 Gb	0.010%	54
Chr3	431 Kb/2.6 Gb	0.016%	47	530 Kb/3.1 Gb	0.017%	45	283 Kb/2.6 Gb	0.011%	52
Chr4	379 Kb/2.4 Gb	0.016%	48	431 Kb/2.7 Gb	0.016%	48	246 Kb/2.4 Gb	0.010%	47
Chr18	149 Kb/0.96 Gb	0.016%	49	178 Kb/1.1 Gb	0.016%	47	96 Kb/0.96 Gb	0.010%	54
Chr19	144 Kb/0.74 Gb	0.019%	44	166 Kb/0.9 Gb	0.018%	44	100 Kb/0.74 Gb	0.013%	48
Chr20	126 Kb/0.78 Gb	0.016%	49	145 Kb/0.92 Gb	0.016%	50	82 Kb/0.78 Gb	0.011%	59
Chr21	63 Kb/0.37 Gb	0.017%	46	85 Kb/0.52 Gb	0.017%	47	42 Kb/0.37 Gb	0.011%	51

Average subread depth = 10

### Comparison with PacBio and Deepconsensus quality scores

Pacbio and deep consensus uses Phred quality score outputs [7, 25] which range from 1 to 93, which corresponds to base call accuracy of 20–99.99999995%. Consensus and polishing algorithms seek to find the correct base as well as assign an accurate assessment of the likelihood of that base being erroneous. To do so, we count mismatches to the reference genome that are not germline variants as errors, but this method overlooks the presence of somatic mutations and considers them as errors. But because our validation dataset is from umbilical cord blood, the quantity of somatic mutations is much smaller than the number of observed mismatches (675 versus 524,575). This gives our validation a theoretical maximum quality value of q80 if the only mismatches we observed were somatic mutations (see supplement).

**Fig. 3:**
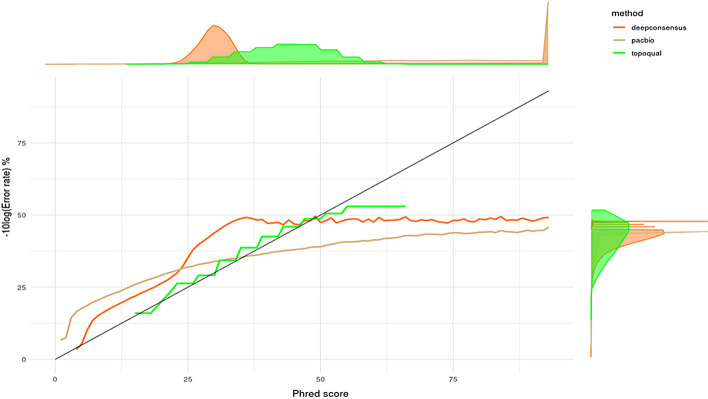
Quality score validation comparison of different methods (expected to fall on the diagonal), chr2. Marginal plots show the distribution

Figure 3 illustrates the algorithm-provided base quality scores (X-axis) compared to the corresponding actual base qualities from analyzing the mismatches in chromosome 2 of the validation dataset (Y-axis). The two marginal plots represent density distribution of the base counts.

At lower quality levels, both PacBio and DeepConsensus exhibit fewer errors than anticipated, but at higher quality levels, both surpass the expected error rates. PacBio reaches a maximum quality of 46, while DeepConsensus achieves 49. Our method, TopoQual, generates quality scores that align with the actual error numbers at both lower and higher quality levels, reaching a maximum of 54.

The distribution of quality scores in PacBio and DeepConsensus is predominantly concentrated around the maximum value, 93. However, the actual measured quality is well below 93. TopoQual more accurately measures the validated quality scores which are roughly normally distributed as expected. Despite a broader range of quality scores in TopoQual, the count of high-quality (> 45) bases is equivalent to that of PacBio (± 1%).

## Conclusion

Correcting errors and providing accurate quality scores is necessary for single molecule sequencing somatic mutation calling. We introduce topoqual, a method for improving consensus sequence accuracy and dramatically increasing the validity of quality values. Topoqual corrects 31.9% of errors vs the PacBio consensus and produces accurate quality scores that have been validated versus a sample with exceedingly low somatic mutation burden. We show that existing methods highly overestimate the quality values of a majority of bases. Statistical methods overestimate base accuracy because of their assumption of total independence of subread sequences. Our quality values validate up to q59 or an error rate of 1e10^−5.9. This work will support the ability to accurately call somatic variants even when only one read samples the somatic variant.

## Supplementary Information

Additional file1

## Data Availability

The datasets analysed during the current study are available in the SRA repository, SRA biosample accession PRJNA1128051. Project name: Topoqual. Project home page: https://github.com/lorewar2/TopoQual. Operating system(s): Linux, MacOS. Programming language: Rust, Python. Other requirements: Rust 1.65.0 + , Python 3.9 + , samtools, pytorch, numpy, pysam. License MIT license. Any restrictions to use by non-academics: None.

## Abbreviations

Mb: Mega base
Kb: Kilo base
Gb: Giga base
POA: Partial order alignment
SUB: Substitution
DEL: Deletion
MAX Q: Maximum quality
